# Why Do We Talk To Ourselves?

**DOI:** 10.1007/s13164-020-00487-5

**Published:** 2020-06-05

**Authors:** Felicity Deamer

**Affiliations:** Institute for Forensic Linguistics, School of Languages and Social Sciences, Aston University, Aston Triangle, Birmingham, B4 7ET UK

## Abstract

Human beings talk to themselves; sometimes out-loud, other times in inner speech. In this paper, I present a resolution to the following dilemma that arises from self-talk. If self-talk exists then either, (i) we know what we are going to say and self-talk serves no communicative purpose, and must serve some other purpose, or (ii) we don’t know what we are going to say, and self-talk does serve a communicative purpose, namely, it is an instance of us communicating with ourselves. Adopting (i) was the strategy taken by Bart Geurts, who claims that the primary purpose of self-talk is to entrain commitments, and is not (primarily) communicative. While accepting that self-talk can usefully play this role, I criticise the view that entraining commitments is self-talk’s fundamental role. I argue that adopting the view that we are self-blind, at least to a significant degree, means that we can accept that self-talk does play a communicative role.

## Introduction

Human beings talk to themselves; sometimes overtly, at other times covertly in what is sometimes called “inner speech”. Call this general phenomenon of talking to oneself, whether overtly or covertly, “self-talk.” Self-talk is not just about talking explicitly to yourself; the first-person (or indeed second-person) pronoun need not feature.^1^ It *can* be explicitly self-directed (“You’re such an idiot!” or “Don’t forget to get some milk”) but it doesn’t have to be (“This meal is so delicious!”). Of course, exactly the same applies when we are talking to others.

My central question is: “What is the purpose of self-talk?”. Vygotsky (1934/1987) saw inner speech as the end result of a developmental process – interpersonal linguistic exchanges (such as those had between the child and a caregiver) become internalized as a “conversation” with oneself, and this internalization of linguistic communication is used to regulate behavior (Fernyhough 2010; Luria 1965; Vygotsky 1934/1987). A large body of empirical work has emerged relating to inner speech, examining its role in self-regulation of cognition and behavior in both children and adults, suggesting that inner speech dysfunction may play a role in psychiatric conditions and developmental disorders involving atypical language skills or deficits in self-regulation (Diaz and Berk 1992; Fernyhough 1996; Vygotsky 1934/1987). In spite of this, several popular accounts of communication don’t allow for the possibility of the speaker and hearer being the same person i.e. when the recipient of a communicative utterance is in fact also the producer of the utterance. This is because of a central, and very reasonable, assumption that communication involves, at least in part, the passing of information from one agent to another. Agent A “has” that information, and agent B lacks it. The puzzling thing about self-talk becomes the problem that one agent cannot both simultaneously *have* and *lack* a given piece of information.

## Gricean Accounts of Communication

One popular account of communication is the Gricean account (Grice 1957). Speakers have communicative intentions, and hearers infer the meaning of utterances on the basis of recognizing those intentions. The Gricean account, like all standard accounts, has an informational disparity between producer and recipient. A has information that B lacks. However, the main Gricean innovation is to make that information primarily information about A’s mind, about A’s communicative intention. B may well glean information about the outside world, but only indirectly, via, for example, the recognition that the utterance was a sincere and literal assertion referring to a recognizable chunk of reality, and a trust that A is knowledgeable about this matter.

Since Grice, there have many variants of this picture (e.g. Horn (1989, 2004), Levinson (2000), and Sperber and Wilson 1995. My primary interest is with this broad family of views. The precise details are unimportant for my current purpose - what matters, and what these views all have in common if they are to count as relevantly “Gricean” (Strawson 1964; Searle 1969, for example, are Gricean in the relevant sense), is that when agent A communicates with agent B, agent A is primarily in the business of giving agent B information about her mind, which agent A has, and agent B lacks.

## The Problem of Self-Talk

The aforementioned assumption about an informational differential seems very reasonable. However, the existence of self-talk presents us with the following inconsistent triad:

1. Self-talk exists.
2. We know our communicative intentions.
3. What we say in self-talk conveys information to an interlocutor (in the case of self-talk, ourselves) about our communicative intentions.

At least one of these three propositions has to be rejected. In short, we have a trilemma. Since we will assume that self-talk does exist, what we have in reality is a dilemma: you have to deny either 2 or 3. Call this dilemma, the Problem of Self-Talk.

## Geurts’ Speech Act Solution

Geurts (2018) also notices the Problem of Self-Talk. His solution is to accept 2, but deny 3. The existence of self-talk makes sense if the role of self-talk is not (primarily) to convey information. If that is not its role, then what is it? Geurts’ answer: to entrain certain commitments.

Geurts argues that, as well as informing others, another purpose of speaking in general is to coordinate actions via commitments (whether privately to oneself or socially to another individual), and this is how it is possible to provide a unified theory of self-talk and “normal” (interpersonal) talk directed at someone else. When talking to others you’re typically informing them *and* entraining commitments. Since in self-talk, you can’t be doing the former, you’re only doing the latter.

What does Geurts mean by entraining commitments? Geurts draws on Speech Act Theory (Austin 1962; Searle 1969). Speech acts are performed against a background of certain social norms. If you break those norms, then you have performed a *bad* speech act. This applies obviously to inter-personal interaction, but the idea is that it has force intra-personally as well.

Geurts claims that different types of speech acts correspond to different types of commitment. Here are some examples.

### Promises

“I’ll mow the lawn”

The speaker makes a commitment to herself to carry out an action.

### Statements (Assertions)

“It snowed in April”

The content of the commitment is a possible state of affairs in the past, but the speech act constrains the speakers’ future actions: The speaker is committed to act in the future on the premise that it snowed in April i.e. to inform people of that fact, to consider this fact when booking a holiday etc.

### Questions

“Shall I go to the shop?”

The speaker becomes committed to the goal of committing himself either to going to the shop or not going to the shop. Self-addressed questions serve to coordinate reasoning processes, and to prompt them to be carried out, and conclusions drawn immediately.

### Orders (Directives)

“Walk the dog!”

The speaker’s goal is that he walks the dog, and the speaker commits himself to that goal.

Orders resemble promises in that they create commitments on the part of the speaker.

In sum, Geurts’ solution to the Problem of Self-Talk is that the purpose of self-talk is not (since it *cannot* be) informational; it must instead be to entrain commitments. This is an ingenious solution and there is much that I like about it. However, it is not entirely without its worries.

## Some Worries for Geurts’ Solution

The first worry is as follows. Although the observation that speech acts entrain commitments is insightful, it seems to have a *limitation of scope*. In other words, it seems to work better for some speech acts than others. For example, the idea that promises and directives are about entraining commitments seems very plausible. However, it seems like a bit of a stretch for questions and assertions. Self-directed questions can be practical or theoretical. Compare “Shall I pick up the kids?” with “What time do I need to pick up the kids?” Certainly the former may entrain commitments in precisely the way that Geurts describes, but the latter seems more plausibly about prompting recall than about eliciting commitments to action. Moreover, when considering self-directed assertions such as “This meal is horrible”, there doesn’t seem to be a plausible motivation to commit to act in the future on the basis of this premise.

The second worry is more serious and it is that Geurts’ solution doesn’t seem to get to the heart of the matter in the intended manner. Just because self-talk *can* be used to entrain commitments, it doesn’t mean that that is *why self-talk emerged in the first place*. One hypothesis worth considering, which is consistent with all of Geurts’ insightful observations about speech acts and commitments, is that self-talk emerged for a more primitive purpose, but gets co-opted for commitment entrainment.

At this point, a clarification is in order. The claim is not one about temporal sequence in either evolution or development. It’s not that self-talk emerged first without commitment entrainment. Although that could well be the case, the claim is one about explanatory primacy. Even if commitment entrainment is there from the outset, the relevant question is: *Why is it through self-talk that commitments are entrained?* Couldn’t they just be entrained by some more purely mental event? In other words, commitment entrainment in itself is not obviously a satisfying explanation for the (mere) existence of self-talk.

## An Alternative Solution

Recall the inconsistent triad:

1. Self-talk exists.
2. We know our communicative intentions.
3. What we say in self-talk conveys information to an interlocutor (in the case of self-talk, ourselves) about our communicative intentions.

My suggestion is: could we retain 3, and drop 2 instead? We typically don’t know our communicative intentions before we speak. This could be the result of a rather localized self-ignorance, specific to language production, or it could be something more global. The stronger, global, claim seems worthy of exploration.

In general, the stronger claim would go, we lack direct access to our mental states. What we have here is a specific case of this general point. Just as we don’t know our own mental states until they make themselves manifest to us via some medium on which we have a grip, so we don’t know our communicative intentions, until we have spoken.

This general claim is very much in keeping with accounts of self-knowledge put forward by Carruthers (2009, 2011) and Cassam (2011). On these views, we never have direct access to our mental states; it always proceeds via *interpretation*. This view allows for interpretation of sensations, emotions, actions, and, of course speech. Indeed, something like my proposed solution is almost directly derivable from the following passage:

All speech – whether the speech of oneself or someone else – needs to be interpreted before it can be understood. Unless we beg the point at issue and assume that subjects have direct introspective access to their own articulatory intentions, the language-comprehension system will need to get to work on the utterance in the normal way. (Carruthers 2009, p.5)

What does this look like as an account of self-talk in particular? Well, in stark contrast to Geurts’s view, where the informational role of self-talk is denied, and some other purpose for it must be found, self-talk (and its more socially acceptable version, *inner speech*) is vital for generating self-knowledge of even rather mundane kinds. One is reminded here of E.M. Forster’s quote, “How can I tell what I think, until I see what I say?”

Of course, as was mentioned earlier, this is perfectly consistent with the idea that self-talk, like any talk, *can* be used to entrain commitments. Furthermore, it addresses the question of why these must be entrained via self-talk (or something sufficiently similar), since – as the person who is both entering into, and holding yourself to, said commitment – it is vital that you know that the commitment has been entered into.

## Addressing Three Concerns

I see some concerns with the position as presented by Carruthers above, and recognise that this calls for modification. After detailing the issues I see with Carruthers’ account, I will put forward my own account, which builds on that of Carruthers. On Carruthers’ view, the communicative intention (the thought/intention/belief) underlying an utterance is something that you are not conscious of until you engage in self-talk, and (“hearing” yourself) then you infer about yourself that you had that intention. But here are three problems with this.

### Are we in Total Ignorance? What about when we Mis-Speak?

It stretches plausibility to claim that we are in total ignorance of what we are trying to say until we say it. For example, we routinely have a sense of what we are trying to say and are quick to correct ourselves when we mis-speak, or otherwise express ourselves inadequately. Surely the simplest explanation of this is that we know our communicative intentions and our actual linguistic performance has failed to fulfil those intentions.

Carruthers does have the resources to deal with this. Recall that processes of interpretation (and hence self-knowledge) can use a variety of input, including sensation, emotion and context too. The fact that you can express yourself badly, or that something can be on the tip of your tongue, is precisely a self-interpretation based on a wide dynamic patchwork of sensation and affect, including the context leading up to the assertion. You know that you meant to ask for a top-up to you glass, because that’s what you do when your glass is empty, you feel thirsty, you want the Champagne etc.

### Do we Always Need to Interpret ourselves when we Speak?

Isn’t our speech usually a reflection of our self-knowledge rather than something that, if we react to it in the right way (namely, interpret it) can generate self-knowledge?

This is perhaps more problematic. What it seems to highlight are two very different ways of thinking about knowledge. One is about explicit declarative knowledge: Information that is stored more or less statically in the mind. The other is more dispositional, dynamic and skills-based. Our everyday practices of knowledge attribution tend to track the latter at least as much, if not more, than the former. It would certainly be strange and misleading to say that someone doesn’t know something simply because they would need to go through some process to make that knowledge manifest. As many have pointed out, you know that Paris is the capital of France, even if you need to think about it for a moment, and you know it, even when you are fast asleep. Similarly, you know your communicative intention even if you haven’t explicitly reflected on what you were saying. You were too busy focusing on your interaction with your interlocutor. That hardly warrants an attribution of self-ignorance. We typically grant people a high degree of epistemic authority over what they say, because people in general are pretty skilled at speaking their minds.

### Does all of our Self-Talk Involve Communicative Intention?

Some self-talk might involve the recognition of one’s own communicative intentions. In other words, in these instances somebody is acquiring self-knowledge by communicating with themselves. But there might, in addition, be more primitive forms of self-talk that don’t work on this communicative (and hence communicative intention) model. They might involve more direct expression.

The sense of “expression” at play is behaviour that reveals, rather than describes (whether intentionally or otherwise), the state that the organism is in. Some linguistic behaviour can be expressive in precisely this sense. Saying “Ouch!” *reveals* that I am in pain, it doesn’t *describe* me as in pain (unlike saying “I’m in pain” does). Similarly, saying “To hell with you!” reveals that I am unhappy with what you’ve done, it doesn’t describe my unhappiness (unlike saying “I’m unhappy with what you’ve done”). Similarly say to yourself “Come on!”, or “I’m such an idiot!”, or whatever, reveals something about you. It seems that a great deal of self-talk is expressive in this sense. Indeed, it’s often this kind of self-talk that manifests itself overtly, that tends not to stay safely locked in the realm of “inner speech”. Of course, if the story I’m proposing is that self-talk enables us to access our own communicative intentions, then what about self-talk that doesn’t function in this way?

On Carruthers’ account we hear our own self-talk and recognise our own communicative intention on the basis of it. This may be the case in some self-talk, but in some other cases of self-talk (whether in inner or private speech), what we are doing is, in the first instance, expressing ourselves, and, when we pay attention to it, revealing our mental states to ourselves. We aren’t, in these cases, communicating with ourselves in the robust Gricean sense. We are rather signalling to ourselves – non-intentionally (although not “by mistake” either!) revealing our own state of mind to ourselves.

Of course, what we can also do is gain intentional control over our expressive vehicles. This way, we get expressive communication; a skilled harnessing of expression. This is not an intention to communicate, not a mere revelation, but an intentional (even if unconscious) revelation of a state of mind. A great deal of ecological human speech likely occupies this middle ground.

What I would say to this is that the Carruthers-inspired picture needs to be embellished.^2^ Contrary to Geurts, self-talk is about informing ourselves, but it is not always, or even predominantly, about inferring our own communicative intentions. Sometimes it is a matter of directly showing ourselves to ourselves.

## Final Remarks

The very existence of self-talk presents us with a dilemma. We can either, as Geurts has recently done, think of self-talk as playing a non-informative role. On his account it entrains commitments instead. This seems unlikely to explain the very existence of self-talk, and doesn’t seem to capture all instances of it. I argue here that an alternative is to drop proposition 2, and argue that self-talk is informative because we are, to some extent, self-blind. Carruthers has a view like this that can only account for instances of self-talk in which we intend to communicate something to ourselves. I argue that it can be embellished and built upon to include more primitive, expressive, forms of self-talk, where you show yourself to yourself. This account of why self-talk exists is not simply that, however. It potentially re-conceptualises the whole relationship between language and thought. Contrary to many views of communication, there is no thought “in mind”, present and accessible, that is then articulated in language. There are simply various exteroceptive and interroceptive sensory states that guide and contextualize the production of linguistic behavior. Often this is for others, but sometimes it is for ourselves.

On the view I have put forward here, what guides our self-speaking is typically not prior knowledge of what we’re going to say, but rather a vaguer sense, prospectively, of what we’re trying to achieve by speaking given the context, and, retrospectively, whether we have expressed ourselves aptly or not once we have spoken. And while we’re actually speaking, it feels (I would suggest) like the exercise of some intangible skill. Sometimes, it must be admitted, we do know exactly what we are going to say. However, when we *do*, it is because we have already gone through this process in a rehearsed episode of inner speech first, that itself was this intangible skill, reaching into the void. Conscious episodes of (linguistic) thinking on this view would simply be acts of speaking, whether in inner speech or out loud. As a result, when people say “I’m just thinking out loud” that is best understood entirely literally.

## Footnotes

1 You can talk explicitly to yourself using either the first or second person (“I can do it!” vs. “You can do it!”); an interesting observation in itself that fits nicely with what I’ll say here.

2 Although I argue that Carruthers’ view has the resources to incorporate the embellishments I have put forward here, the notion that self-knowledge could be gained via expression seems to be inconsistent with his broader epistemological leanings (Carruthers 2011: 84–89).
